# Suboxone: History, controversy, and open questions

**DOI:** 10.3389/fpsyt.2022.1046648

**Published:** 2022-10-28

**Authors:** Andy Sivils, Paige Lyell, John Q. Wang, Xiang-Ping Chu

**Affiliations:** Department of Biomedical Sciences, School of Medicine, University of Missouri-Kansas City, Kansas City, MO, United States

**Keywords:** Suboxone, buprenorphine, addiction, opioid use disorder, opioid epidemic, medication-assisted treatment, harm reduction, stigma

## Abstract

There are more than 200 opioid overdose deaths each day in the US. In combating this epidemic we look to available treatment tools. Here, we find only three medications approved by the Food and Drug Administration (FDA) for the treatment of opioid use disorder. Of the three, buprenorphine is of particular importance due to its reduced overdose potential as a partial opioid agonist. Evidence supports its clinical equivalence to its full agonist cousin methadone, and suggests that it is better slated for long-term treatment of opioid use disorder compared to the non-selective opioid antagonist naltrexone. Buprenorphine is most popularized within Suboxone, a medication which also contains the non-selective opioid antagonist naloxone. The naloxone has no additional effect when the drug is taken as instructed, as it is intended to prevent diversion in those that would attempt to inject the medication. While Suboxone is regarded by some as the future of medical treatment, others have expressed concerns. This review aims to explore the history, controversy, and open questions that surround buprenorphine and its most prescribed variation, Suboxone. These include its pharmacological, legislative, and social history, alternative indications, efficacy as a treatment of opioid use disorder, and more. Armed with this information, the reader will have a more in-depth and holistic understanding of the medication’s place in their community.

## Introduction

In 2021, drug overdose deaths in the United States exceeded 107,000–a record high (1). 80,816 of these involved opioids (1). These totals are a substantial increase from just 2 years prior in 2019, where an estimated 70,630 lives were lost to overdose and 49,860 of them were opioid-related (2, 3). Looking back further, since the beginning of the opioid epidemic in 1999, there has been a nine-fold increase in opioid-involved overdose deaths (1, 4). These statistics reveal a pattern over the last two decades worthy of investigation.

The Centers for Disease Control and Prevention (CDC) identifies three waves of the opioid epidemic: first, prescription opioids (e.g., oxycodone, hydrocodone); second, heroin; and third, synthetic opioids (e.g., fentanyl). The first wave began shortly after OxyContin (oxycodone controlled-release) was approved by the Food and Drug Administration (FDA) in 1995. By 1999, some 400,000 Americans admitted to abusing oxycodone (2, 4, 5). Just 4 years later, this number increased to nearly 2.8 million Americans, and by 2010, it rose to nearly 12 million (5, 6). The number of opioid prescriptions and the rate of opioid-overdose deaths increased in parallel, both showing a four-fold increase from 1999 to 2010 (7, 8). This first wave of deaths was a surprise to prescribers who had been told that the drug’s controlled-release mechanism made it less susceptible to abuse.

As the population of prescription drug users rose, so did the price. Users had to find more economic alternatives. Thus, the second wave centered around heroin–a cheap, more potent substitution to prescription opioids (2). In 2010, heroin overdose deaths had a sharp uptick which resulted in a 350% increase by 2013 (2–4, 9). During this wave, the American Society of Addiction Medicine (ASAM) reported that about 80% of new heroin users had first started their illicit drug use by misusing prescription opioids (8). But as time went on, and the market for illicit opioids continued to increase, even more potent drugs began to take the lead.

The third wave was therefore marked by the surge in synthetic opioids, most commonly Fentanyl, shortly after 2014 (2, 4). According to the National Forensic Laboratory Information System, law enforcement reports of Fentanyl increased by 247% from the second half of 2013 to the first half of 2014 (10). Today, synthetic opioids account for over 80% of all opioid overdose deaths (11). Fentanyl is 50 times more potent than heroin and 100 times more potent than morphine (12). The problem was so bad during this period that the CDC attributes the general decrease in life expectancy from 2014 to 2017 to the increase in these opioid overdose deaths (13). For perspective, this was the first decline in general life expectancy since 1993–when the acquired immunodeficiency syndrome (AIDS) epidemic ravaged the US (13). Now, more than 150 Americans die every day due to synthetic opioid overdose (12).

Using the three-wave categorization is useful to understand the way this epidemic has evolved and worsened overtime. However, it’s equally important to recognize that with each new wave the old waves do not die out. They simply plateau and are surpassed by a new medium. All together, they demonstrate a serious problem in our community and an increasing risk to a vulnerable patient population. The medical field’s attempts at reducing these deaths have not all been a success.

Detoxification, abstinence-based programs, and psychosocial therapies alone have consistently failed (14–16). Currently, the FDA recommends medication-assisted treatment (MAT) for substance use disorders (SUD) (17). For opioid use disorder (OUD) specifically, there are three medications approved by the FDA–an opioid antagonist, naltrexone, and two opioid agonists, methadone and buprenorphine (17). Each of these forms of MAT has been shown to significantly benefit OUD patients, most notably by decreasing the rates of overdose and death (14, 17–21). One meta-analysis found that the risk of death due to overdose in patients receiving no MAT was eight times higher than in those receiving MAT (19). Prior research has also shown that MAT leads to a reduction in the rates of HIV and hepatitis C transmission, relapse, and criminal activity (14, 16, 20, 21).

Choosing the appropriate medication is multifactorial, and treatment should always be individualized. However, MAT with the use of an opioid agonist is generally preferred especially in those with moderate-to-severe OUD (17). The largest barrier to MAT with naltrexone is that a patient must undergo full detoxification before initiating treatment (18, 21). While buprenorphine and methadone can be started as soon as a patient has made the decision to seek treatment, with some consideration to not precipitate withdrawal depending on the patient’s current use (18, 21).

Withdrawal from short-acting opioids (e.g., heroin, fentanyl) can last four to ten days (22). During this time, a patient is prone to experience insomnia, diarrhea, intense pains, and anxiety resulting in severe distress (20, 22). Therefore, many patients struggle to fully detox prior to the start of treatment and ultimately choose to return to their illicit drug of choice (18). That said, evidence suggests that naltrexone has similar efficacy in the treatment of OUD compared to the opioid agonists when successfully administered (18). However, the data regarding overdose and death following relapse demonstrate that the opioid antagonist is worse compared to buprenorphine and methadone (19, 22, 23). During abstinence from opioids, as in naltrexone therapy, there is a loss of opioid tolerance–a tolerance that is for the most part maintained on agonist medications (22). Therefore, patients who relapse after antagonist medication use are more likely to misjudge the amount of drug they can safely use.

Another significant deficit of naltrexone compared to the agonist medications is the lack of a recommendation for treatment in pregnant women. With a significant rise in OUD prevalence seen in this population, this is of particular importance to the medical community (24, 25). One study found that from 2010 to 2017, there was a 131% increase in OUD in pregnant women documented at the time of birth (24). Another study found that from 1999 to 2013, the incidence of neonatal abstinence syndrome increased nearly 300% (26). Opioid use during pregnancy has been linked to poor outcomes in the infant such as birth defects (e.g., cleft palate/lip, atrial and ventral septal defects, spina bifida), low birth-weight, increased utilization of healthcare services during infancy, and sudden infant death syndrome (27–29). It is worth noting that the prevalence of OUD has also significantly increased in women of childbearing age; thus, opioid agonists may be more appropriate for this patient population as well (21, 22, 25, 30).

As for the two agonist medications that can be given to women in this population, methadone was the first to be approved and was the first successful medication used to treat OUD (17). It was originally approved by the FDA in 1947 for its long-acting analgesic and antitussive effects (31). However, in the early 1960’s, Dr. Vincent Dole and Dr. Marie Nyswander began the first clinical studies to examine its potential as an OUD treatment (31). They hypothesized that the drug’s long-acting, morphine-like effects would satisfy opioid cravings and decrease drug-seeking behavior in patients addicted to heroin and morphine (31). This provides patients with a chance to lead more productive, fulfilling lives. The results of these studies quickly gained popularity, and methadone’s indication for OUD was approved in 1972 (31). The influence methadone had on OUD treatment even landed it a spot on the World Health Organization’s List of Essential Medicines (20, 32).

Now, there are three agencies that oversee methadone regulation at the federal level–the FDA, the National Institute of Drug Abuse (NIDA), and the Substance Abuse and Mental Health Services Administration (SAMHSA) (31). These regulations ensure methadone is only prescribed to patients who are currently inpatient or part of a federally regulated treatment program (31). In addition to these three regulatory bodies, methadone treatment is further restricted at the state level and sometimes at the county level as well (31). Though some have argued to the contrary, many contend that this level of oversight is needed. This is partly explained by previous attempts at maintenance therapy for OUD. Morphine clinics, which were mainly for soldiers who had become addicted following war wounds, proved to cause more harm than good (31). Following the Harrison Narcotic Act of 1914, the American Medical Associated (AMA) adopted a resolution in 1920 (31). This resolution led to a closing of all the “ambulatory clinics” that were providing maintenance morphine therapy to addicts–considering these venues to not be medicine but instead simple drug-trafficking (31).

Some 50 years later, with the Nixon administration in full swing, legislation was passed which led to the creation of drug schedules (31). With the creation of these, the newly formed Drug Enforcement Administration (DEA) labeled methadone as a Schedule II substance due to its high potential for abuse and dependency (20, 31, 33, 34). Shortly after the implementation of methadone therapy in the 1970s, reports of diversion began to emerge (35). This, coupled with shifting presidential administrations and government sentiment toward addiction treatment, led to continued legislative changes regulating the prescription of methadone maintenance (31).

From then on, many patients have had difficulties gaining access to the medication. For example, treatment centers which can prescribe methadone are often too far from patients’ homes and are not offered in every state (36, 37). This is especially true for patients living in rural areas, who have historically been disproportionately affected by deaths due to opioid overdose (36, 38). Furthermore, treatment centers typically have long waitlists, which puts a strain on patients simply trying to live their lives as productive members of society (37). Regardless of these hurdles, methadone represented a shift in the way the US combatted OUD and served as a harbinger for treatment efforts to come.

The mainstay of contemporary treatment is the second of the two agonists, buprenorphine. From 2004 to 2011, the use of buprenorphine increased 2,318% compared to methadone’s 37% (39). Its abuse which led to emergency room visits rose by 384% over the same period compared to methadone’s 48% (39–41). Buprenorphine’s pharmacological activity is unique compared to methadone and other pure agonists. Additionally, its side effect profile is better for patients than other opioids (42). These factors led to its inclusion in the medication Suboxone, a 4:1 formulation with the opioid antagonist naloxone (43). Due to these special features and the growing sentiment that it is the gold standard for OUD management, the modern era of medical care hinges on its safety, efficacy, and validity as a treatment.

**FIGURE 1:**
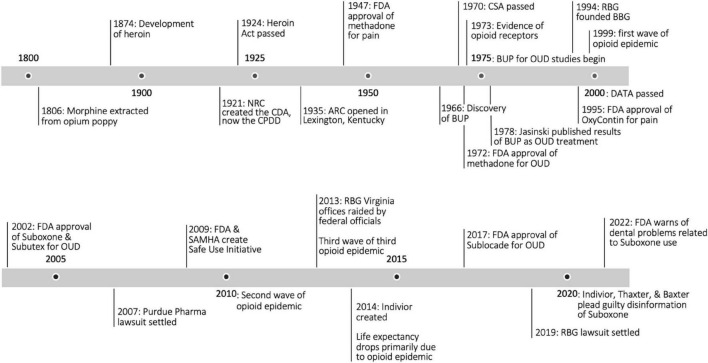
Opioid timeline. NRC, National Research Council; CDA, Committee on Drug Addiction; CPDD, Committee on Problems of Drug Dependence; ARC, Addiction Research Center; FDA, Food and Drug Administration; BUP, buprenorphine; CSA, Controlled Substance Act; OUD, opioid use disorder; RBG, Reckitt Benckiser Group; BBG, Buprenorphine Business Group; DATA, Drug Addiction Treatment Act; SAMSHA, Substance Abuse and Mental Health Services Administration (2, 5, 13, 31, 43, 45, 47, 48, 55).

The objectives of this review are to investigate Suboxone’s history (Figure 1), relevant controversy, differing schools of thought, research gaps, and to provide an overview of the state-of-the-art treatment of opioid use disorder with this medication. It is the hope of the authors that each reader acquires a deeper appreciation for the nuance of this medication and a greater sense of responsibility in treating those with addiction–not as problems to be solved, or deaths to be prevented, but as lives to be saved.

## Pharmacological history and controversy

The opium poppy *Papaver somniferum* is thought to date back to around 5,000 BC, making it one of the oldest known medicinal plants (44). It was not until 1,806 when morphine was first extracted from this plant. In the 1,850s, shortly after the invention of the hypodermic needle, morphine use dramatically increased because it was found to be effective agent for treating post-operative pain and as an adjunct to minor surgical procedures (45, 46). The injection of morphine was found to be particularly useful during the American Civil War, where it was extensively used to treat wounded soldiers (44). However, this led to many opioid-dependent veterans returning home after the war. In fact, this was so prominent that opioid addiction was referred to as “the soldiers’ disease” (44).

In 1874, heroin was synthesized (47). It was marketed as less addictive than other opioids and was thus recommended over the use of morphine. Prescription heroin was given for analgesia, cough-suppression, and to treat morphine addiction (47, 48). Fifty years after being synthesized, the Heroin Act determined that the medicinal use of heroin is illegal (48). It is interesting to look back on this era, with the knowledge of contemporary science, and see the clear mistakes that were made. But it is important to recognize that with the information professionals had at the time, it seemed a reasonable course of action. One sizeable deficit in their knowledge was the fact that opioid receptors had not been delineated yet.

It was not until the 1950s when researchers first proposed the idea of opioid receptors, and it was not until the 1970s that researchers were able to provide evidence of their existence (46, 47). We now know that there are three major opioid receptor families–mu (MOR), kappa (KOR), and delta (DOR). Each family consists of at least two to four subtypes; however, to avoid complexity, this review will focus on each opioid receptor family as a single entity (49). Each of these families consists of G protein-coupled receptors (GPCRs) which are primarily, but not exclusively, found in the peripheral and central nervous system as they function to inhibit pain pathways and regulate reward circuitry (45, 50). These families are in part distinguished by their distinct physiological effects, in addition to their varying specificities for endogenous and exogenous opioid ligands (Table 1; 45, 51, 52). Analyzing these receptors’ interactions with buprenorphine will help us establish an understanding of the molecule and its unique place in pharmacological history.

**TABLE 1:** Opioid receptor families, their major functions, and associated endogenous agonists (52, 53).

Receptor family	Mu	Kappa	Delta
Functions	Analgesia Euphoria Physical dependence Respiratory depression Reduced gastric motility	Analgesia Dysphoria Reduced gastric motility	Analgesia Tolerance Anxiolytic
Endogenous agonists	Endorphins	Dynorphins	Endorphins

Buprenorphine is generally accepted to be a partial agonist at the MOR based on *in vitro* assays used for its FDA approval; however, there is debate regarding the appropriate label describing the drug’s pharmacological activity (54–57). According to Trevor et al., a partial agonist produces less than full effect at maximum dosage compared to a full agonist (52). Conceptually, medical providers will explain this by saying that the molecule binds to the receptor but stays bound for a longer duration, therefore blocking other molecules from binding. Multiple studies have shown that buprenorphine does exactly this as it binds at MORs with high affinity and exhibits slow dissociation (58). MOR activation decreases pain perception and increases reward-related behavior, as evidenced by experiments with MOR-knockout mice which demonstrated increased pain perception and decreased reward-related behaviors following morphine administration (50, 51, 59). Thus, buprenorphine activity at the MOR receptor decreases pain and increases reward-related behavior up to the purported ceiling of these effects.

Furthermore, the longer duration of receptor occupancy is reported to lead to less severe withdrawal symptoms and reduction in cravings compared to other opioids (58). Perhaps an even more clinically relevant difference is the risk of overdose. When a patient on buprenorphine takes an opioid such as heroin or fentanyl, the risk of overdose is significantly decreased because there are less receptors available to be bound (58, 60). These characteristics have led this chemical to the center of the world’s conversation on substance use and treatment.

However, there are other explanations of the pharmacology which conflict with this description. These arise first when reviewing clinical efficacy. The primary desired clinical effect of opioids is analgesia. Multiple studies have shown that buprenorphine can produce the same or greater analgesic effects as other opioid agonists (56, 57, 61, 62). This suggests that whatever ceiling effect buprenorphine might have at the MOR, it is not low enough to make a difference in the perception of pain–or maybe something other than partial agonism happens at this receptor (56, 57, 61, 62).

One claim is that buprenorphine is actually a full agonist at the mu receptor (56). The studies which led to the conclusion that buprenorphine was a partial agonist came from the aforementioned *in vitro* assays in the 1970s (56). To the contrary, more modern evidence suggests that not only is there no ceiling effect on analgesia produced by agonist activity at the mu receptor, but that buprenorphine is on average 30 times more potent than morphine (56, 61). Another report involving a group of physicians and scientists from all over the world concluded that buprenorphine clearly behaves as a full mu-opioid agonist for analgesia in clinical practice (57). They only mentioned its ceiling effect, or proposed partial agonist status, in regard to the ceiling on respiratory depression (57).

Some researchers contend that even if this molecule had partial agonist activity beyond its influence on respiratory depression, because the desired clinical effect–analgesia–is produced when only 5–10% of mu receptors are occupied such a classification would be inaccurate (56, 63). This idea is important for understanding the differing pharmacological schools of thought. Imagine a full agonist and partial agonist provide a hypothetical number of relief points. The full agonist can provide ten times more relief points than the partial agonist. Suppose it is then discovered that the relief points required for subjective pain relief are well below the maximum that the full agonist can provide. In fact, the amount required is below even the partial agonist’s maximum. Then the hypothesized pharmacological difference in these medications would be irrelevant. Thus, it is “nowadays widely accepted” in the scientific community that buprenorphine behaves as a full agonist in analgesia at the MOR (64).

But the arguments continue to widen in scope. The previously mentioned inhibition of other opioids due to buprenorphine partial agonist activity at the mu receptor has not been consistently produced. As shown in a randomized, double-blinded, four-arm trial study by Oifa et al., when buprenorphine was added to morphine for pain control in the 12 h following abdominal surgery, the analgesia was superior and not inhibited (56, 65). A similar study reported equivalent findings where subcutaneous administration of buprenorphine led to a synergistic effect with the participant group receiving the medication in addition to morphine (56, 66). The previously cited consensus expert report found once again that buprenorphine administration with other opioids created no problems in producing the needed analgesia (57). Together, these findings make a compelling case for buprenorphine’s status a full agonist at the MOR. But others contend that instead of full and partial agonism, perhaps another kind of pharmacology is taking shape.

This third option involves what is called biased agonism. This is a relatively new pharmacological receptor theory which involves the activation of a specific downstream pathway compared to others after G-protein coupled receptor (GPCR) activation by a ligand (64, 67, 68). In the case of the MOR, there are two main pathways after GPCR activation: G-protein or arrestin mediated downstream pathways (67). Research has shown that in arrestin-3-knockout mice (also known as β-arrestin-2), the respiratory depressant and gastrointestinal effects of morphine are reduced (67, 69). An investigation into the bias of buprenorphine reported a G-protein bias (70). These findings underscore the mu agonist activity, apart from respiratory depression, and provide a more accurate pharmacological description of the substance. But even this description could be incomplete, the other two opioid receptor families may play a part.

Unlike most mu-opioid agonists, buprenorphine acts as an antagonist at KORs and DORs. The KOR family is unique in its production of dysphoria and depression (49). Endogenously, this is produced by dynorphins (51). It is thought that this pathway helps regulate the MOR/endorphin pathway (51). Thus, KOR antagonism has been shown to offer some improvement in comorbid depression and anxiety (49, 58, 71). Together with buprenorphine’s known action on the receptor, it is hypothesized that buprenorphine’s anti-hyperalgesia is due to its KOR antagonism (64). There is also some evidence suggesting that the antagonism of DORs may reduce the addictive profile of the drug.

A further look at these receptors and the reward system yields some consensus conclusions. For one, the MOR family produces euphoria and is highly implicated in rewarding behavior (53). It is also known that the DOR family acts similarly with MORs to increase analgesia (72). When MORs and DORs are activated, they cause a synergistic increase in dopamine in the nucleus accumbens, which results in more reward (i.e., reinforced addictive behaviors) (53, 72, 73).

In contrast, KOR agonism decreases dopamine in the nucleus accumbens when activated (73). Experiments on adolescent rats showed decreased social play after kappa agonist administration (74). Thus, KOR antagonism leads to an increase in dopamine in the nucleus accumbens relative to its physiologic or synthetic antagonism (75). In the case of buprenorphine, this means that both the mu agonism and kappa antagonism produce rewarding effects in known circuity, and it is possible that the delta antagonism produces a comparatively negative effect. No current science can quantify the exact reward, namely dopamine, produced by each substance due to the variation in receptor density, ligand composition, individual neuronal variance, and many other technological factors. This is a notable gap in the research which can only be filled with considerable time, effort, and scientific advancement. However, the known receptor theory and accompanying data are sufficient to support the notion of a rewarding pharmacology for buprenorphine, like other opioids–not constituted by partial agonism.

Regardless of the school of thought one falls in given the evidence, arguments for and against its status as a partial agonist are weakened by the fact that all opioids produce differing analgesic effects in patients and in animal models (47, 76). Pain is hard to measure. It is a subjective entity that contains an emotional component. Investigations have shown that perception of pain does not always correlate to the intensity of a pain stimulus (76). So, the mainstay of clinical measurement in this class of medication is quantitatively hard to gauge, thus limiting the validity of any conclusive statements. And regardless, it is widely accepted that it produces less adverse effects like respiratory depression and euphoria (60, 61, 77, 78). Thus, buprenorphine is still clinically relevant given its superior side effect profile compared to other agonists.

Outside of the pharmacology of buprenorphine is the one other component of Suboxone: naloxone. This mu-opioid antagonist counteracts opioid agonists, but only when intramuscularly, subcutaneously, intranasally, or intravenously administered (79). When given sublingually, as in Suboxone, naloxone has no effect on buprenorphine and is not intended to. The addition of naloxone was an effort to prevent diversion and abuse of Suboxone *via* the previously listed mechanisms. It does not reduce or effect the agonist activity of buprenorphine when Suboxone is taken as prescribed. Essentially, a patient administering Suboxone as instructed is taking buprenorphine alone.

However, there has been recent debate if naloxone is actually efficacious in Suboxone when diversion is attempted (60). According to Suboxone’s FDA label, the half-life of naloxone ranges from 2 to 12 h, while that of buprenorphine ranges from 24 to 42 h (55). The antagonist effects of naloxone would theoretically wear off long before the agonism of buprenorphine (60, 80). Even still, some evidence suggests that the subjective effect of a drug is not the driving purpose for addiction. Instead, the drug-associated rewards could be entirely separated from the pleasurable effects of the drug, where individuals pursue largely subconscious manifestations and are sensitized over repeated drug exposures (60, 81–84). And while the pharmacology itself has been disputed, some clarity may be found within the broader history of the molecule. Clinicians and scientists can garner important insight into the origins of varying schools of thought regarding the proper use of the medication.

## History of medical policy and controversy

In 1921, the National Research Council (NRC) created the Committee on Drug Addiction (CDA), eventually known as the Committee on Problems of Drug Dependence (CPDD), in order to find morphine substitutes as a way to approach the “opium problem” (43). In 1935, the first US Narcotic Farm opened in Lexington, Kentucky (43). The 1,500 bed institution was shared with the Federal Bureau of Prisons as the subjects for human experimental research were felons convicted of drug charges (85). It would later become known as the Addiction Research Center (ARC) (43). John Lewis, a chemist in the UK who worked at a home products company named Reckitt and Colman (Reckitt), discovered buprenorphine in 1966 out of an effort to develop analgesics (43). He later helped supply buprenorphine to the ARC *via* Reckitt and Colman for research purposes (43). Lewis describes his work developing buprenorphine by saying “we were trying to beat morphine, not methadone” (43, 86).

The scientific community’s emphasis on buprenorphine as a candidate for addiction treatment came in the wake of bureaucratic reshaping that occurred in 1975 (43, 86). The National Academy of Sciences reorganized its structure which led to the termination of the Committee on Problems of Drug Dependence (CPDD) as an NRC committee (43). Then, in 1976 the Federal Bureau of Prisons decided to phase out all participation of ARC’s prison recruitment channel (43). In defense of the program, researchers placed emphasis on buprenorphine as a sign of progress in developing alternatives to methadone maintenance (43). Subsequently, in 1978, the landmark paper displaying research from ARC on the therapeutic potential of buprenorphine for addiction was published (87). However, the journey from scientific data to being sold as a medication was filled with hurdles.

One of the hurdles seemed to be inherited from its MAT predecessor methadone, regulation by federal authorities citing diversion risk (43). Another set of hardships involved scheduling, both nationally and internationally. Charles O’Keefe, a former Clinton advisor and CEO of Reckitt Benckiser Pharmaceuticals, Inc. (eventually absorbed into Indivior) from 1991–2004, was a notable figure in defending buprenorphine’s classification internationally (43). In an interview with historian Nancy Campbell, O’Keefe said “you had to jointly defend the class of drugs, to keep the agonist/antagonist where they were” (43). At the national level things loosened on buprenorphine, as it was changed from schedule II to schedule V in 1985 (43, 88).

Even Reckitt had their doubts at first. Mirroring the attitude of most analgesic pharmaceutical distributers, they worried their product might be “tainted” from the perspective of providers and patients if it was also used in addiction treatment (43). This reluctance was amplified, as remembered by Chris Chapleo the Director of Buprenorphine Business from 2012–2014 for Reckitt, by the “misuse, abuse, (and) diversion of buprenorphine, the analgesic product” (43). In fact, more than half of Temgesic, the buprenorphine analgesic medication, was being used off-label to treat addicts in France (43). This led to it being included in the Convention on Psychotropic Substances in the late 1980s (43). After that, there was a 50% drop in sales (43).

More than a decade after the seminal paper displaying buprenorphine’s potential as an addiction therapeutic the US government placed a focus on finding an addiction treatment. Specifically, the NIDA, which ARC was absorbed into, aimed at relapse prevention and had buprenorphine in its sights (85). As a result of this keen interest, a deal was ratified in 1994 between Reckitt and the US government (43). Reckitt negotiators were purportedly convinced by arguments about “social responsibility” toward drug addicts and the ethics of withdrawing buprenorphine from patients taking it for pain (43).

NIDA pledged to co-fund the development of Suboxone for US distribution in the treatment of addiction (43). Orphan status for Suboxone and Subutex was offered on the rare grounds of the cost recovery principle–that Rickett might not be able to make back its cost (43, 89). This granted the company 7 years of monopoly on the US market. After a series of NIDA funded trials and lobbying efforts, the Drug Addiction Treatment Act passed in 2000 (43). Physicians could now treat opiate-dependent persons in the outpatient setting following an 8-h certification (43). Then, in 2002, FDA approval was granted for Subutex and Suboxone (43). Less than two decades later, recouping the costs was never in doubt. In fact, Suboxone generated $785 million in net revenue in 2019 compared to Viagra’s roughly $500 million (90, 91).

But the DEA did end up restricting buprenorphine more, taking it from schedule V to schedule III in the same year the FDA approved Suboxone (43, 88). Citing deaths from overdose and thousands of documented cases of diversion, the agency contended that the medication was not an accurate fit for schedule V (43, 88). Federal and state governments have intervened with Suboxone since then, but this information can be more thoroughly reviewed elsewhere (92–96). The scheduling change, lawsuits, and other regulatory restrictions are believed by some treatment professionals to be reflections of systemic forces. The same kind that has affected the treatment of addiction for its entire history–stigma.

The US National Sigma Studies have collected data *via* face-to-face interviews since 1996. Analysis of the most recent data revealed the first significant reduction in stigma for one mental health diagnosis, major depression (97). However, the same data revealed that the idea that alcohol dependence is related to bad character or moral failure has increased over the same period (97). Similarly, one recent paper aimed at investigating the barriers to medication assisted treatment for opioid use disorder identified stigma as a notable problem (98). In fact, there is stigma specific to buprenorphine which involves seeing the medication as “a crutch” and goes further to assert that the person using that medication is not really sober (99).

These forces have a significant impact on individuals with substance use disorders who are in recovery and are thus vitally important to the medical community tasked with treating them. Due to the increasing negative influence on this specific patient population and their standard of care one researcher, Madden, interviewed 47 addiction treatment professionals and examined stigma specific to MAT, including Suboxone (100). One of the addiction treatment professionals reported running into friction when calling in a prescription for Suboxone, underlining negative sentiments like “it’s a crutch drug”, “you’re keeping them addicted to something else”, and “it’s a money making scheme” (100). They reported calling the pharmacy multiple times, only to be told the prescription was never sent and suspected that the pharmacists preferred not to have these patients at their business (100). This suggests a unique feature of intervention stigma compared to conventional condition stigma, the patient is not the only one targeted.

Providers are also stigmatized for engaging in MAT. Some of this can be attributed to inexperience with addiction science, where some health professionals may not have extensive training or experience with treating this patient population (100). However, the paper also suggests that some of intervention stigma might be from other treatment professionals themselves, particularly those that support an abstinent only approach. It seems there are two major claims from this camp which contest the tenants of MAT. The first claim being that you do not need MAT to live a successful and drug free life (100). This serves as a defense of patients from providers who otherwise might push an unnecessary medication. The second claim being that even if MAT is helpful and legitimate as medical care, extended treatment duration might verge on negligence. Many treatment professionals are proponents of these viewpoints, others are distinctly opposed, and some are somewhere in the middle.

Another of the interviewed addiction treatment professionals used an analogy in defense of MAT which stated “if you have the flu, you do take medicine, but not forever” (100). According to the study author, this was an example of intervention stigma in the sense that it viewed drugs like Suboxone as medicine but not as a medicine that can be taken for extended periods of time (100). Another study followed up on Madden’s findings with similar evidence of intervention stigma in providers who saw MAT as a temporary tool, one that should be weaned off as soon as possible in order to achieve the goal of being “drug free” (101). Outside of formal treatment professionals there are collectives like the 12-step community which are fundamental in many recovering patients’ lives. These groups sometimes express ideas like “you are not clean until you are not on anything,” insinuating that the use of MAT is not truly sober and according to Madden perpetuate intervention stigma (102). There has not been a consensus among treatment providers whether these ideas are evidence of stigma or are valid opposing arguments on how to best care for those with substance use disorders.

## Discussion

There are no signs that addiction is going away. It continues to invade communities all around the world, stealing lives 1 day at a time. Efforts have been made to curtail this phenomenon, specifically as it relates to opioid use disorder. Heroin was generated as a treatment for morphine addiction, methadone for heroin, and now buprenorphine. If the available data accurately represent the current state of the epidemic, providers and scientists working to care for patients do not have a grip on this issue. However, progress has been made.

For one, we know that buprenorphine has a safer side effect profile than all the other opioid agonist medications. Specifically, the deadliest element of opioid overdose is the decrease in respiratory drive and buprenorphine leads to less respiratory depression than the other opioid agonists (39). This fact, along with the benefit of no longer using illicit opioids which can contain undetermined amounts of fentanyl, may account for the almost ten-fold decrease in risk of death while on the medication compared to being out of treatment (103).

A more recent systematic review published in JAMA Psychiatry found that the all-cause mortality during either methadone or buprenorphine maintenance was less than half the rate while not in treatment (104). This same study demonstrated that people with opioid dependence were at a substantially lower risk of suicide, cancer, drug-related, alcohol-related, and cardiovascular-related mortality during their opioid agonist therapy compared to outside their therapy (104). These findings are among many which demonstrate the unequivocal truth–opioid use disorder patients are protected from death by buprenorphine treatment.

However, these same studies highlight something else too. Discontinuing opioid agonist therapy is deadly. In fact, the risk of death increases by more than three times following cessation of buprenorphine (105). In the first 4 weeks following cessation, all-cause mortality jumps more than six times higher (104, 105). The first multisite, large randomized controlled trial (RCT), also published in JAMA Psychiatry, found that more than 90% of patients returned to illicit drug use after discontinuation (106). Compounding this risk is the fact that most patients cycle in and out of substitution treatment (107, 108). Patients are thus exposed to high-risk periods repeatedly.

One of the only long-term opioid agonist studies reported between 9 and 15% of participants still received treatment 5 years after their first dose (107). A buprenorphine specific study that examined over a thousand patients from 2002–2014 found that only one fourth of participants had a period of 5 years or more in treatment (109). That number dropped to 5.2% when looking for a ten-year treatment period (109). This suggests that roughly 95% of patients started on buprenorphine will discontinue treatment over a ten-year period. But what about tapering the dose? A recent RCT published in JAMA showed that even tapering the dose is associated with increased risk for overdose and mental health crisis (110).

If a clinician were to survey the available data and make their patient care decisions based on the state-of-the-art evidence-based medicine, they would place every OUD patient on Suboxone and never taper or take them off it. That is what data suggests is the most efficacious treatment course to protect the patient from death and adverse events. Some would suggest that the only force fighting against long-term MAT in the form of Suboxone are those displaying intervention stigma (100). They contend that most of the resistance to MAT is born out of ignorance, a lack of education. “The absence of harm reduction science in training sets the stage for a professional treatment community rife with intervention stigma due to limited knowledge of the full spectrum of evidence-based recovery approaches” (100). If an educated treatment professional with personal experience in recovery from a substance use disorder is encountered that disagrees with the logic behind harm reduction, or simply with MAT itself, Madden contends that “simply showing results of rigorous research may not change the minds of counselors who place value on their own experiences and 12-step community narratives.” But maybe there are scientific grounds for further investigation.

With the rigorous research displayed here, one could fairly conclude that buprenorphine treatment only works if it is used continually. That most patients unfortunately will not remain in treatment continually and will be repeatedly exposed to a higher risk of overdose. Specifically, a 2021 JAMA psychiatry paper that included more than 26,000 patients in opioid agonist therapy found a crude mortality rate of 54.19 per 1,000 in the 4 weeks following opioid agonist therapy (104). This is higher than the mortality rate of a patient with OUD in the general healthcare system, found to be 48.6 per 1,000 (111). No experimental evidence is required to conclude that the licit use of opioids does not differ neurochemically from the illicit use of opioids. Legalized buprenorphine molecules behave the same as illegal buprenorphine molecules. The receptors in the brain which help to process opioids are not aware of medically approved substances versus those that are not.

This line of thinking seems to suggest that opioid substitution is replacing one drug with another drug. Many treatment professionals would contend that this suggestion is a display of ignorance, of stigma. Here, listening to the developers of medications like buprenorphine can provide deeper insight. One of the fathers of the CPDD, who helped conceptualize the strategy of finding a morphine replacement, maintained that “a substance which will support and maintain the “addicted state” is essentially addictive in and of itself” (112). Dole, the first clinical researcher to study the potential medical benefit of opioid substitution with methadone, contended that a “significant number of patients through methadone management have attained a reasonable degree of social rehabilitation. Their dependence has not been ameliorated, it has not been treated, it may have been augmented, but the patient and society have gained” (113). Medication-assisted treatment is not a treatment of the disease that afflicts the patient, it is intended to reduce the negative impact that substance use disorders have on society and provide the patient with a chance to participate in society as a law-abiding citizen.

This is not a bad thing; in fact, it demonstrates significant progress in how society interacts with those that suffer from addiction and provides patients with a suitable option if they wish to discontinue their illicit drug use. Though it is necessary to note that some researchers see this differently. One author contends that “methadone maintenance constitutes a qualitative shift from penal to medical control, one that purports to be more humanistic and benevolent, but one that replaces one form of “domination” and “incarceration” with another” (114). Is this a stigmatic response? Or is it a logical conclusion drawn in conjunction with the founding members of opioid substitution?

Here, many will fall back to the consensus statement that addiction, and thus opioid use disorder, is a chronic illness. Some patients with diabetes may need insulin for the rest of their life–some patients with opioid use disorder may need Suboxone for the rest of their life. If their medical professional is following empiric evidence, they will insist that they do use Suboxone and that they never taper or get off it. And what would be the issue here? Other than an antiquated idea that some inherent value exists in the latent neurochemistry of an unmedicated brain–a stigma. But again, the unfortunate gaps in modern research prevent any strong conclusions, and good science requires us to challenge our assumptions.

Is the brain equivalent to the pancreas? Do vessels embattled by cholesterol, rescued by a statin, equate to the conscious experience of craving which leads to relapse? No. They do not. The analogy that addiction is a chronic disease was helpful in shedding much of the bigotry that contended addiction to be a moral failing. But here we must push ourselves to look beyond this helpful sentiment toward the reality of this disease. It pervades the deepest level of consciousness a person has. It nestles underneath it, quietly steering its victim to the next use again and again. It manifests itself not as the seizures from alcohol withdrawal, or as the constipation in sustained opioid use, but as the suburban teenager who decides one more pill will not hurt them. The young professional who ties off their arm in the parking lot of their job, hiding beneath the complex rationalization that they are more productive at work. The Suboxone patient who begins to contemplate calling his old dealer after he discontinued treatment. The decades long MAT patient who fears missing just one dose. This disease does not manifest in blood sugar levels, it manifests in thought. It is not a patient’s progressive insulin resistance; it is a subtle shift in their perception of the world. That pain is not a hemoglobin A1c, or worsening neuropathy. It is the unspeakable heartache as a patient helplessly administers the next dose, licit or illicit, that they desperately wish they did not have to take. These are the threads sewn into the very fabric of the patient’s existence.

So, if we are to dispense the lifesaving medication that is Suboxone, we must holistically review the evidence with the patient. We are not treating your disease; we are making it easier to perform your roles in life. We are not freeing you from the bondage of your illness; we are shifting your dependence from an illicit substance to a licit one–which has less chance of killing you. We are not telling you that you cannot be free from drug dependence; we are following evidence-based medicine which insists that you should take this opioid for the rest of your life. Perhaps this is the best that we can do as a field, perhaps it is not.

## Author contributions

All authors listed have made a substantial, direct, and intellectual contribution to the work, and approved it for publication.

## References

[1] CDC. *U.S. overdose deaths in 2021 increased half as much as in 2020 – but are still up 15%.* Atlanta, GA: CDC (2022).

[2] CDC. *Understanding the epidemic drug overdose CDC injury center.* Atlanta, GA: CDC (2022).

[3] Mattson CL. Trends and geographic patterns in drug and synthetic opioid overdose deaths — United States, 2013–2019. *MMWR Morb Mortal Wkly Rep.* (2021) 70:202–7. 10.15585/mmwr.mm7006a4 33571180 PMC7877587

[4] CDC. *Opioid data analysis and resources CDC’s response to the opioid overdose epidemic CDC.* Atlanta, GA: CDC (2022).

[5] FDA. *Timeline of selected FDA activities and significant events addressing opioid misuse and abuse.* Silver Spring, MD: FDA (2022).

[6] Robinson SM Adinoff B. The classification of substance use disorders: Historical, contextual, and conceptual considerations. *Behav Sci Basel Switz.* (2016) 6:E18. 10.3390/bs6030018 27548233 PMC5039518

[7] Hedegaard H Minino A Warner M. *Products – data briefs – number 394 – december 2020.* Atlanta, GA: CDC (2020).

[8] Rudd RA Seth P David F Scholl L. Increases in drug and opioid-involved overdose deaths — United States, 2010–2015. *MMWR Morb Mortal Wkly Rep.* (2016) 65:1445–52. 10.15585/mmwr.mm655051e1 28033313

[9] National Institute on Drug Abuse. *Overdose death rates.* Bethesda, MD: National Institute on Drug Abuse (2022).

[10] Special Report. *Opiates and related drugs reported in NFLIS, 2009-2014.* Washington, DC: Special Report (2022).

[11] CDC. *Death rate maps & graphs drug overdose CDC injury center.* Atlanta, GA: CDC (2022).

[12] CDC. *Fentanyl Facts.* Atlanta, GA: CDC (2022).

[13] Kochanek K Anderson R Arias E. *Products – health E Stats – changes in life expectancy at birth, 2010–2018.* Hyattsville, MD: National Center for Health Statistics (2020).

[14] Velander JR. Suboxone: Rationale, science, misconceptions. *Ochsner J.* (2018) 18:23–9. 29559865 PMC5855417

[15] Srivastava A Kahan M Nader M. Primary care management of opioid use disorders: Abstinence, methadone, or buprenorphine-naloxone? *Can Fam Physician.* (2017) 63:200–5. 28292795 PMC5349718

[16] National Institute on Drug Abuse. *National institute on drug. How effective are medications to treat opioid use disorder?.* Bethesda, MD: National Institute on Drug Abuse (2022).

[17] Substance Abuse and Mental Health Services Administration. *Medications for opioid use disorder (treatment improvement protocol (TIP) series 63). Report No.: PEP21-02-01–002.* Rockville, MD: Substance Abuse and Mental Health Services Administration (2021). 332 p.

[18] Xxxx. Research briefs. *Pharm Ther.* (2018) 43:59–60.

[19] Ma J Bao YP Wang RJ Su MF Liu MX Li JQ Effects of medication-assisted treatment on mortality among opioids users: A systematic review and meta-analysis. *Mol Psychiatry.* (2019) 24:1868–83. 10.1038/s41380-018-0094-5 29934549

[20] WHO. *Clinical guidelines for withdrawal management and treatment of drug dependence in closed settings.* Geneva: World Health Organization (2009). 26269862

[21] Connery HS. Medication-assisted treatment of opioid use disorder: Review of the evidence and future directions. *Harv Rev Psychiatry.* (2015) 23:63–75. 10.1097/HRP.0000000000000075 25747920

[22] Schuckit MA. Treatment of opioid-use disorders. *N Engl J Med.* (2016) 375:357–68. 10.1056/NEJMra1604339 27464203

[23] Degenhardt L Larney S Kimber J Farrell M Hall W. Excess mortality among opioid-using patients treated with oral naltrexone in Australia. *Drug Alcohol Rev.* (2015) 34:90–6. 10.1111/dar.12205 25302627

[24] Hirai AH Ko JY Owens PL Stocks C Patrick SW. Neonatal abstinence syndrome and maternal opioid-related diagnoses in the US, 2010-2017. *JAMA.* (2021) 325:146–55. 10.1001/jama.2020.24991 33433576 PMC7804920

[25] Mack KA Jones CM Paulozzi LJ. Vital signs: Overdoses of prescription opioid pain relievers and other drugs among women — United States, 1999–2010. *Morb Mortal Wkly Rep.* (2013) 62:537–42. 23820967 PMC4604783

[26] Ko JY Patrick SW Tong VT Patel R Lind JN Barfield WD. Incidence of neonatal abstinence syndrome — 28 States, 1999–2013. *MMWR Morb Mortal Wkly Rep* (2016) 65:799–802. 10.15585/mmwr.mm6531a2 27513154

[27] Lind JN Interrante JD Ailes EC Gilboa SM Khan S Frey MT Maternal use of opioids during pregnancy and congenital malformations: A systematic review. *Pediatrics.* (2017) 139:e20164131. 10.1542/peds.2016-4131 28562278 PMC5561453

[28] Ko JY Yoon J Tong VT Haight SC Patel R Rockhill KM Maternal opioid exposure, neonatal abstinence syndrome, and infant healthcare utilization: A retrospective cohort analysis. *Drug Alcohol Depend.* (2021) 223:108704. 10.1016/j.drugalcdep.2021.108704 33894458 PMC8893024

[29] Forray A. Substance use during pregnancy. *F1000Research.* (2016) 5:F1000FacultyRev–887. 10.12688/f1000research.7645.1 27239283 PMC4870985

[30] O’Donnell FT Jackson DL. Opioid use disorder and pregnancy. *Mo Med.* (2017) 114:181–6. 30228577 PMC6140233

[31] Institute of Medicine (Us) Committee on Federal Regulation of Methadone Treatment Rettig RA Yarmolinsky A. *Federal regulation of methadone treatment.* Washington, D.C: National Academies Press (1995). 25121195

[32] WHO. *WHO model list of essential medicines – 22nd list, 2021.* Geneva: World Health Organization (2021).

[33] Whelan PJ Remski K. Buprenorphine vs methadone treatment: A review of evidence in both developed and developing worlds. *J Neurosci Rural Pract.* (2012) 3:45–50. 10.4103/0976-3147.91934 22346191 PMC3271614

[34] National Institute on Drug Abuse. *What is the treatment need versus the diversion risk for opioid use disorder treatment?.* Bethesda, MD: National Institute on Drug Abuse (2021).

[35] Inciardi JA. *Methadone diversion: Experiences and issues.* Bethesda, MD: National Institute on Drug Abuse (1977). 124 p. 10.1037/e473742004-001

[36] Pasman E Kollin R Broman M Lee G Agius E Lister JJ Cumulative barriers to retention in methadone treatment among adults from rural and small urban communities. *Addict Sci Clin Pract.* (2022) 17:35. 10.1186/s13722-022-00316-3 35841076 PMC9284487

[37] Welsh C Valadez-Meltzer A. Buprenorphine. *Psychiatry Edgmont.* (2005) 2:29–39. 21124750 PMC2994593

[38] Keyes KM Cerdá M Brady JE Havens JR Galea S. Understanding the rural–urban differences in nonmedical prescription opioid use and abuse in the United States. *Am J Public Health.* (2014) 104:e52–9. 10.2105/AJPH.2013.301709 24328642 PMC3935688

[39] Schiller EY Goyal A Mechanic OJ. *Opioid overdose.* Treasure Island, FL: StatPearls Publishing (2022). 29262202

[40] Substance Abuse and Mental Health Services Administration. *Drug abuse warning network, 2011: National estimates of drug-related emergency department visits. HHS Publ No SMA 13-4760.* Rockville, MD: Substance Abuse and Mental Health Services Administration (2013). 100 p.

[41] Substance Abuse and Mental Health Services Administration. *Center for behavioral health statistics and quality. The DAWN report: Emergency department visits involving buprenorphine.* Rockville, MD: Substance Abuse and Mental Health Services Administration (2013). 7 p. 27606401

[42] Khanna IK Pillarisetti S. Buprenorphine – an attractive opioid with underutilized potential in treatment of chronic pain. *J Pain Res.* (2015) 8:859–70. 10.2147/JPR.S85951 26672499 PMC4675640

[43] Campbell ND Lovell AM. The history of the development of buprenorphine as an addiction therapeutic. *Ann N Y Acad Sci.* (2012) 1248:124–39. 10.1111/j.1749-6632.2011.06352.x 22256949

[44] Schiff PL. Opium and its alkaloids. *Am Jorunal Pharm Educ.* (2002) 66:186–94.

[45] Dhaliwal A Gupta M. *Physiology, opioid receptor.* Treasure Island, FL: StatPearls Publishing (2022). 31536249

[46] Brownstein MJ. A brief history of opiates, opioid peptides, and opioid receptors. *Proc Natl Acad Sci.* (1993) 90:5391–3. 10.1073/pnas.90.12.5391 8390660 PMC46725

[47] Pasternak GW Pan YX. Mu opioids and their receptors: Evolution of a concept. *Pharmacol Rev.* (2013) 65:1257–317. 10.1124/pr.112.007138 24076545 PMC3799236

[48] Manchikanti L Sanapati J Benyamin RM Atluri S Kaye AD Hirsch JA. Reframing the prevention strategies of the opioid crisis: Focusing on prescription opioids, fentanyl, and heroin epidemic. *Pain Physician.* (2018) 21:309–26. 10.36076/ppj.2018.4.309 30045589

[49] Lalanne L Ayranci G Filliol D Gavériaux-Ruff C Befort K Kieffer BL Kappa opioid receptor antagonism and chronic antidepressant treatment have beneficial activities on social interactions and grooming deficits during heroin abstinence. *Addict Biol.* (2017) 22:1010–21. 10.1111/adb.12392 27001273 PMC5590636

[50] Darcq E Kieffer BL. Opioid receptors: Drivers to addiction? *Nat Rev Neurosci.* (2018) 19:499–514. 10.1038/s41583-018-0028-x 29934561

[51] Feng Y He X Yang Y Chao D Lazarus LH Xia Y. Current research on opioid receptor function. *Curr Drug Targets.* (2012) 13:230–46. 10.2174/138945012799201612 22204322 PMC3371376

[52] Trevor AJ Katzung BG Kruidering-Hall M. *Katzung & trevor’s pharmacology: Examination & board review.* 11th ed. New York, NY: McGraw-Hill Medical (2015). 586 p.

[53] Wang S. Historical review: Opiate addiction and opioid receptors. *Cell Transplant.* (2019) 28:233–8. 10.1177/0963689718811060 30419763 PMC6425114

[54] Drug Approval Package. *Subutex (buprenorphine HCI) suboxone (buprenorphine HCI & naloxone HCI dihydrate) NDA #020732 & 020733.* Silver Spring, MD: FDA (2004).

[55] Indivior Inc. *Full prescribing information for suboxone sublingual tablets for sublingual administration CIII.* Silver Spring, MD: FDA (2018).

[56] Giron SE Lai G Griffis CA Zhang SJ. Demystifying buprenorphine with current evidence-based practice in acute and chronic pain management. *AANA J.* (2022) 90:225–33. 35604865

[57] Pergolizzi J Aloisi AM Dahan A Filitz J Langford R Likar R Current knowledge of buprenorphine and its unique pharmacological profile. *Pain Pract Off J World Inst Pain.* (2010) 10:428–50. 10.1111/j.1533-2500.2010.00378.x 20492579

[58] Walton MR Bruce RD. Clinical pearls for buprenorphine treatment. *Prim Health Care.* (2021) 11:362.

[59] Mechling AE Arefin T Lee HL Bienert T Reisert M Ben Hamida S Deletion of the mu opioid receptor gene in mice reshapes the reward–aversion connectome. *Proc Natl Acad Sci.* (2016) 113:11603–8. 10.1073/pnas.1601640113 27671662 PMC5068324

[60] Gudin J Fudin JA. Narrative pharmacological review of buprenorphine: A unique opioid for the treatment of chronic pain. *Pain Ther.* (2020) 9:41–54. 10.1007/s40122-019-00143-6 31994020 PMC7203271

[61] Raffa RB Haidery M Huang HM Kalladeen K Lockstein DE Ono H The clinical analgesic efficacy of buprenorphine. *J Clin Pharm Ther.* (2014) 39:577–83. 10.1111/jcpt.12196 25070601

[62] Yassen A Olofsen E Romberg R Sarton E Danhof M Dahan A. Mechanism-based pharmacokinetic–pharmacodynamic modeling of the antinociceptive effect of buprenorphine in healthy volunteers. *Anesthesiology.* (2006) 104:1232–42. 10.1097/00000542-200606000-00019 16732095

[63] Davis MP. Twelve reasons for considering buprenorphine as a frontline analgesic in the management of pain. *J Support Oncol.* (2012) 10:209–19. 10.1016/j.suponc.2012.05.002 22809652

[64] Infantino R Mattia C Locarini P Pastore AL Maione S Luongo L. Buprenorphine: Far beyond the “ceiling.”. *Biomolecules.* (2021) 11:816. 10.3390/biom11060816 34072706 PMC8230089

[65] Oifa S Sydoruk T White I Ekstein MP Marouani N Chazan S Effects of intravenous patient-controlled analgesia with buprenorphine and morphine alone and in combination during the first 12 postoperative hours: A randomized, double-blind, four-arm trial in adults undergoing abdominal surgery. *Clin Ther.* (2009) 31:527–41. 10.1016/j.clinthera.2009.03.018 19393843

[66] Chen LH Nemirovsky A Gong QY. Interaction of combined administration of intrathecal morphine with subcutaneous morphine or buprenorphine. *Acta Pharmacol Sin.* (2000) 21:685–9. 11501175

[67] Conibear AE Kelly EA. Biased view of μ-opioid receptors? *Mol Pharmacol.* (2019) 96:542–9. 10.1124/mol.119.115956 31175184 PMC6784500

[68] Violin JD Crombie AL Soergel DG Lark MW. Biased ligands at G-protein-coupled receptors: Promise and progress. *Trends Pharmacol Sci.* (2014) 35:308–16. 10.1016/j.tips.2014.04.007 24878326

[69] Raehal KM Walker JKL Bohn LM. Morphine side effects in beta-arrestin 2 knockout mice. *J Pharmacol Exp Ther.* (2005) 314:1195–201. 10.1124/jpet.105.087254 15917400

[70] Burgueño J Pujol M Monroy X Roche D Varela MJ Merlos M A Complementary scale of biased agonism for agonists with differing maximal responses. *Sci Rep.* (2017) 7:15389. 10.1038/s41598-017-15258-z 29133887 PMC5684405

[71] Lalanne L Ayranci G Kieffer BL Lutz PE. The kappa opioid receptor: From addiction to depression, and back. *Front Psychiatry.* (2014) 5:170. 10.3389/fpsyt.2014.00170 25538632 PMC4258993

[72] Chung PCS Kieffer BL. Delta opioid receptors in brain function and diseases. *Pharmacol Ther.* (2013) 140:112–20. 10.1016/j.pharmthera.2013.06.003 23764370 PMC3775961

[73] Lutfy K Cowan A. Buprenorphine: A unique drug with complex pharmacology. *Curr Neuropharmacol.* (2004) 2:395–402. 10.2174/1570159043359477 18997874 PMC2581407

[74] Trezza V Damsteegt R Achterberg EJM Vanderschuren LJMJ. Nucleus accumbens μ-opioid receptors mediate social reward. *J Neurosci Off J Soc Neurosci.* (2011) 31:6362–70. 10.1523/JNEUROSCI.5492-10.2011 21525276 PMC3098965

[75] Bruijnzeel AW. Kappa-opioid receptor signaling and brain reward function. *Brain Res Rev.* (2009) 62:127–46. 10.1016/j.brainresrev.2009.09.008 19804796 PMC2787673

[76] Khera T Rangasamy V. Cognition and pain: A review. *Front Psychol.* (2021) 12:673962. 10.3389/fpsyg.2021.673962 34093370 PMC8175647

[77] Dahan A van Lemmen M Jansen S Simons P van der Schrier R. Buprenorphine: A treatment and cause of opioid-induced respiratory depression. *Br J Anaesth.* (2022) 128:402–4. 10.1016/j.bja.2021.12.001 34996591

[78] Kuo A Wyse BD Meutermans W Smith MT. In vivo profiling of seven common opioids for antinociception, constipation and respiratory depression: No two opioids have the same profile. *Br J Pharmacol.* (2015) 172:532–48. 10.1111/bph.12696 24641546 PMC4292966

[79] Substance Abuse and Mental Health Services Administration. *Naloxone.* Rockville, MD: SAMHSA (2022).

[80] Blazes CK Morrow JD. Reconsidering the usefulness of adding naloxone to buprenorphine. *Front Psychiatry* (2020) 11:549272. 10.3389/fpsyt.2020.549272 33061915 PMC7517938

[81] Fischman MW. Relationship between self-reported drug effects and their reinforcing effects: Studies with stimulant drugs. *NIDA Res Monogr.* (1989) 92:211–30. 10.1037/e468292004-001 2512494

[82] Robinson TE Berridge KC. The neural basis of drug craving: An incentive-sensitization theory of addiction. *Brain Res Brain Res Rev.* (1993) 18:247–91. 10.1016/0165-0173(93)90013-P 8401595

[83] Leyton M Casey KF Delaney JS Kolivakis T Benkelfat C. Cocaine craving, euphoria, and self-administration: A preliminary study of the effect of catecholamine precursor depletion. *Behav Neurosci.* (2005) 119:1619–27. 10.1037/0735-7044.119.6.1619 16420164

[84] Meyer PJ King CP Ferrario CR. Motivational processes underlying substance abuse disorder. *Curr Top Behav Neurosci.* (2016) 27:473–506. 10.1007/7854_2015_391 26475159 PMC4851611

[85] Campbell ND. “A new deal for the drug addict”: The addiction research center, lexington, kentucky. *J Hist Behav Sci.* (2006) 42:135–57. 10.1002/jhbs.20167 16586455

[86] Sontag D. *Addiction treatment with a dark side.* New York, NY: The New York Times (2013).

[87] Jasinski DR Pevnick JS Griffith JD. Human pharmacology and abuse potential of the analgesic buprenorphine: A potential agent for treating narcotic addiction. *Arch Gen Psychiatry.* (1978) 35:501–16. 10.1001/archpsyc.1978.01770280111012 215096

[88] Brown J III. *Schedules of controlled substances: Rescheduling of buprenorphine from schedule V to schedule III.* Springfield, VA: Drug Enforcement Administration (2002).

[89] Wellman-Labadie O Zhou Y. The US orphan drug act: Rare disease research stimulator or commercial opportunity? *Health Policy Amst Neth.* (2010) 95:216–28. 10.1016/j.healthpol.2009.12.001 20036435

[90] Indivior Plc. *FY 2019 Results.* Richmond, VA: Indivior PLC (2020).

[91] Mikulic M. *Pfizer’s Viagra revenue worldwide 2003-2019.* Hamburg: Statista (2022).

[92] New York State Attorney General. *AG James, States Reach Settlement With Reckitt Over Allegations Of Improper Marketing Of Suboxone.* New York, NY: New York State Attorney General (2019).

[93] U.S. Attorney’s Office Western District of Virginia. *Indivior Solutions Sentenced as Part of $2 Billion Resolution of False Safety Claims Concerning Suboxone.* Charlottesville, VA: The United States Attorney’s Office Western District of Virginia (2020).

[94] Department of Justice Office of Public Affairs. *Justice Department Obtains $1.4 Billion From Reckitt Benckiser Group in Largest Recovery in a Case Concerning an Opioid Drug in United States History.* Washington, DC: U.S. Department of Justice (2019).

[95] Department of Justice Office of Public Affairs. *Former Medical Director of Suboxone Manufacturer Indivior Sentenced in Connection With Drug Safety Claims.* (2020). Available online at: https://www.justice.gov/opa/pr/formermedicaldirector-suboxone-manufacturer-indivior-sentenced-connectiondrug-safety#:~:text=Timothy%20Baxter%2C%20the%20former%20medical,marketing%20of%20an%20opioid%20drug (accessed August 27, 2022).

[96] Simpson B. *FDA Warns That Belbuca, Suboxone, and Zubsolv are Associated With Tooth Loss and Other Significant Dental Problems.* (2022). Available online at: https://www.burgsimpson.com/ohio/2022/01/fda-warns-that-belbucasuboxone-and-zubsolv-are-associated-with-tooth-loss-and-other-significantdental-proble ms/ (accessed August 27, 2022).

[97] Pescosolido BA Halpern-Manners A Luo L Perry B. Trends in public stigma of mental illness in the US, 1996-2018. *JAMA Netw Open.* (2021) 4:e2140202. 10.1001/jamanetworkopen.2021.40202 34932103 PMC8693212

[98] Madras BK Ahmad NJ Wen J Sharfstein JS. Improving access to evidence-based medical treatment for opioid use disorder: Strategies to address key barriers within the treatment system. *NAM Perspect.* (2020) 2020:10.31478/202004b. 10.31478/202004b 35291732 PMC8916813

[99] Mackey K Veazie S Anderson J Bourne D Peterson K. Barriers and facilitators to the use of medications for opioid use disorder: A rapid review. *J Gen Intern Med.* (2020) 35(Suppl, 3):954–63. 10.1007/s11606-020-06257-4 33145687 PMC7728943

[100] Madden EF. Intervention stigma: How medication-assisted treatment marginalizes patients and providers. *Soc Sci Med.* (2019) 232:324–31. 10.1016/j.socscimed.2019.05.027 31125801

[101] Dickson-Gomez J Spector A Weeks M Galletly C McDonald M Green Montaque HD. “You’re not supposed to be on it forever”: Medications to treat opioid use disorder (MOUD) related stigma among drug treatment providers and people who use opioids. *Subst Abuse Res Treat.* (2022) 16:11782218221103860. 10.1177/11782218221103859 35783464 PMC9243471

[102] Richard EL Schalkoff CA Piscalko HM Brook DL Sibley AL Lancaster KE “You are not clean until you’re not on anything”: Perceptions of medication-assisted treatment in rural Appalachia. *Int J Drug Policy.* (2020) 85:102704. 10.1016/j.drugpo.2020.102704 32173274 PMC8018539

[103] Dupouy J Palmaro A Fatséas M Auriacombe M Micallef J Oustric S Mortality associated with time in and out of buprenorphine treatment in french office-based general practice: A 7-year cohort study. *Ann Fam Med.* (2017) 15:355–8. 10.1370/afm.2098 28694272 PMC5505455

[104] Santo T Jr. Clark B Hickman M Grebely J Campbell G Sordo L Association of opioid agonist treatment with all-cause mortality and specific causes of death among people with opioid dependence: A systematic review and meta-analysis. *JAMA Psychiatry.* (2021) 78:979–93. 10.1001/jamapsychiatry.2021.0976 34076676 PMC8173472

[105] Sordo L Barrio G Bravo MJ Indave BI Degenhardt L Wiessing L Mortality risk during and after opioid substitution treatment: Systematic review and meta-analysis of cohort studies. *BMJ.* (2017) 357:j1550. 10.1136/bmj.j1550 28446428 PMC5421454

[106] Weiss RD Potter JS Fiellin DA Byrne M Connery HS Dickinson W Adjunctive counseling during brief and extended buprenorphine-naloxone treatment for prescription opioid dependence: A 2-phase randomized controlled trial. *Arch Gen Psychiatry.* (2011) 68:1238–46. 10.1001/archgenpsychiatry.2011.121 22065255 PMC3470422

[107] Bell J Burrell T Indig D Gilmour S. Cycling in and out of treatment; participation in methadone treatment in NSW, 1990–2002. *Drug Alcohol Depend.* (2006) 81:55–61. 10.1016/j.drugalcdep.2005.05.010 15993552

[108] Hser YI Evans E Grella C Ling W Anglin D. Long-term course of opioid addiction. *Harv Rev Psychiatry.* (2015) 23:76–89. 10.1097/HRP.0000000000000052 25747921

[109] Weinstein ZM Kim HW Cheng DM Quinn E Hui D Labelle CT Long-term retention in office based opioid treatment with buprenorphine. *J Subst Abuse Treat.* (2017) 74:65–70. 10.1016/j.jsat.2016.12.010 28132702 PMC5312773

[110] Agnoli A Xing G Tancredi DJ Magnan E Jerant A Fenton JJ. Association of dose tapering with overdose or mental health crisis among patients prescribed long-term opioids. *JAMA.* (2021) 326:411–9. 10.1001/jama.2021.11013 34342618 PMC8335575

[111] Hser YI Mooney LJ Saxon AJ Miotto K Bell DS Zhu Y High mortality among patients with opioid use disorder in a large healthcare system. *J Addict Med.* (2017) 11:315–9. 10.1097/ADM.0000000000000312 28426439 PMC5930020

[112] King MR Himmelsbach CK Sanders BS. *Dilaudid (dihydromorphinone): A review of the literature and a study of its addictive properties.* Washington, D.C: U.S. Government Printing Office (1935). 38 p.

[113] Eddy NB. *The national research council involvement in the opiate problem: 1928-1971.* Washington, D.C: National Academy of Sciences (1973).

[114] Bennett C. Methadone maintenance treatment: Disciplining the ‘addict.’. *Health Hist.* (2011) 13:130–57. 10.1353/hah.2011.0019 22329263

